# Glycoxidised LDL Induced the Upregulation of Axl Receptor Tyrosine Kinase and Its Ligand in Mouse Mesangial Cells

**DOI:** 10.1371/journal.pone.0050297

**Published:** 2012-11-30

**Authors:** Young Sook Kim, Dong Ho Jung, Eunjin Sohn, Junghyun Kim, Jin Sook Kim

**Affiliations:** Korean Medicine-Based Herbal Drug Research Group, Herbal Medicine Research Division, Korea Institute of Oriental Medicine (KIOM), Daejeon, Republic of Korea; Broad Institute of Harvard and MIT, United States of America

## Abstract

**Aim/Hypothesis:**

Low-density lipoprotein (LDL) is subjected to glycoxidation in diabetes, and a novel signalling mechanism by which glycoxidised LDL functions in glomerular mesangial cells remains to be ascertained.

**Methods:**

We performed gene expression analysis in mouse glomerular mesangial cells treated with LDL modified by glycation and oxidation (GO-LDL, 100 µg/ml) for 48 h by using DNA microarray analysis and quantitative real-time PCR. We examined the GO-LDL-specific changes in gene and protein expression in mesangial cells and glomeruli of type 2 diabetic Zucker diabetic fatty (ZDF) rats.

**Results:**

By microarray profiling, we noted that GO-LDL treatment increased Axl receptor tyrosine kinase (Axl) mRNA expression (∼2.5-fold, p<0.05) compared with normal LDL (N-LDL) treatment in mesangial cells. Treatment with GO-LDL also increased the protein levels of Axl and its ligand Gas6 as measured by Western blotting. These increases were inhibited by neutralising Axl receptor-specific antibody. Silencing Gas6 by siRNA inhibited GO-LDL-induced Axl expression in mesangial cells. Axl and Gas6 protein were also increased in cells cultured in high glucose (30 mM) or methylglyoxal (200 µM). Gas6 treatment increased the expression and secretion of TGF-β1 protein, a key regulator of extracellular matrix expression in the glomeruli of diabetic kidneys. Immunohistochemical analyses of glomeruli from 20-week-old ZDF rats exhibited increased Axl protein expression. Rottlerin, a selective PKC-δ inhibitor, completely blocked Gas6-induced TGF-β1 expression.

**Conclusions/Interpretation:**

These data suggest that LDL modified by glycoxidation may mediate Axl/Gas6 pathway activation, and this mechanism may play a significant role in the pathogenesis of diabetic nephropathy.

## Introduction

Diabetic nephropathy (DN) is the most common cause of end-stage renal disease worldwide and is characterised by glomerular basement membrane thickening, mesangial cell expansion and hypertrophy, and the accumulation of extracellular matrix (ECM) components from mesangial cells [1], [2]. Low-density lipoprotein (LDL) has been implicated in diabetic microvascular complications, and modified LDL (including enhanced glycation, oxidation, and glycoxidation) levels are significantly increased in diabetic patients, even those with good glycemic control, compared with the levels in normal subjects [3]. Modified LDL leads to alterations of the apoB protein to the extent that it is no longer recognised by the LDL receptor to regulate cholesterol feedback [4]. This modified LDL is taken up through scavenger receptors, and it comprises foam cells. In addition, it accelerates the development of glomerular injury in diabetes via increased transforming growth factor (TGF)-β1 expression; TGF-β is a key regulator of ECM that triggers the proliferation of mesangial cells in DN. Proliferation of mesangial cells is a hallmark of glomerular disease, and understanding its regulatory mechanism is clinically important [3].

Microarray technology is a tool to elucidate new therapeutic targets for the treatment of diabetes and diabetic microvascular complications [5], [6]. During microarray profiling, we observed that glycoxidised LDL (GO-LDL) increased Axl expression in mesangial cells. In this study, we demonstrate that cells treated with GO-LDL exhibit GO-LDL-specific increases in the expression of Axl and its ligand growth arrest gene 6 (Gas6) via increased TGF-β1 expression and protein kinase C activation. In this article, we demonstrate the novel mechanism by which GO-LDL mediates Axl upregulation and its crosstalk with Gas6, which could be relevant to the pathogenesis of diseases such as DN. Recently, studies demonstrated that Axl plays a role in metastasis as a novel therapeutic target in solid tumours such as metastatic ovarian cancer and breast cancer tumours [7]–[8]. In addition, reports suggest that Axl plays a role in the pathogenesis of vascular and diabetic diseases. Axl receptor tyrosine kinase (Axl) is a ∼140-kDa protein expressed in various cell types, including endothelial cells, vascular smooth muscle cells, and mesangial cells [9]–[11]. Gas6, a ligand for Axl, stimulates mesangial cell proliferation and hypertrophy through binding to its cell-surface Axl receptor [12], [13]. Axl and Gas6 expression are increased in the glomeruli of rats with type 1 diabetes and experimental glomerulonephritis [12], [13]. However, nothing is known regarding the relationship between GO-LDL and Axl/Gas6 signalling pathways in the context of diabetic complications such as DN. In this study, we specifically studied GO-LDL-induced gene expression profile in glomerular mouse mesangial cells (MMCs) *in vitro* using Oligo-GE arrays and real-time qPCR. The molecular mechanism by which GO-LDL mediates the expression of Axl and Gas6 in MMCs under diabetic culture conditions was analysed.

**Figure 1:**
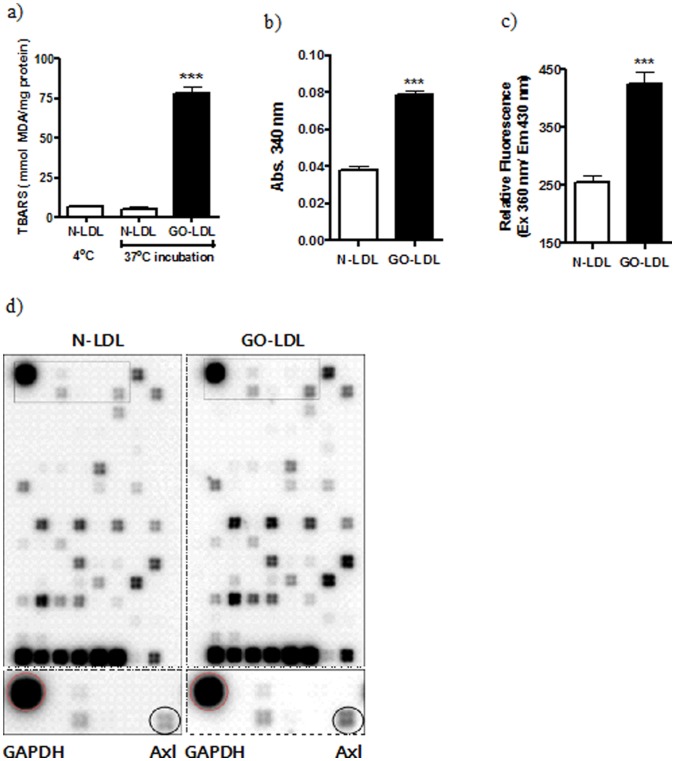
Characterisation of GO-LDL and profiling of genes regulated by GO-LDL in MMCs. (a) TBARS content (marker for peroxidation levels) in GO-LDL was increased compared to that in N-LDL. (b) The degree of glycation was assessed by measuring the amount of lysine residues remaining after modification. The amount of modified-lysine in GO-LDL was significantly increased compared with that in N-LDL (c) Relative fluorescence was increased in GO-LDL compared to that in N-LDL. (d) DNA array profiling shows the differentially expressed genes in response to GO-LDL (right panel) and N-LDL treatment (left panel). All data are expressed as the mean ± S.E.M. (n = 3). ^***^P<0.001 vs. N.

## Methods

### Materials

Human native LDL was obtained from Calbiochem (San Diego, CA, USA). Anti-Axl and anti-TGF-β1 antibodies were purchased from Cell Signaling (Beverly, MA, USA). Anti-Gas6 antibody and mouse TGF-β1 ELISA systems were obtained from R&D Systems (Minneapolis, MN, USA). Dulbecco’s modified eagle’s medium (DMEM)/F-12 and foetal calf serum (FBS) were obtained from Gibco BRL (Grand Island, NY, USA), and enhanced chemiluminescence reagent (ECL) was purchased from GE Healthcare UK Ltd (Buckinghamshire, UK). All other reagents were obtained from Sigma-Aldrich (St. Louis, MO, USA).

**Figure 2:**
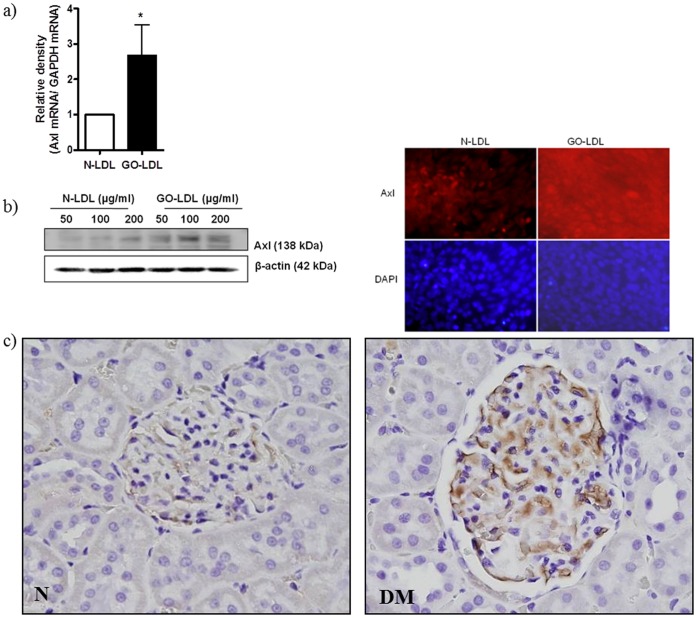
GO-LDL induces Axl mRNA protein expression in MMCs. (a) The bar graph shows the real time-qPCR result of Axl mRNA levels in GO-LDL treated MMCs, and the bottom panel shows the Western blot analyses of Axl protein. All data are expressed as the mean ± S.E.M. (n = 3). ^*^P<0.05 vs. N-LDL. (c) Micrograph of immunochemical analyses of Axl expression in glomeruli from normal (left) and type 2 (right) diabetic rats. Bar graph on the right shows a significant 3-fold induction in Axl protein expression in diabetic glomeruli. All data are expressed as the mean ± S.E.M. (n = 4). ^**^P<0.01 vs. N.

### *In vitro* Modification and Characterisation of Modified LDL

Human native LDL (N-LDL; Calbiochem, San Diego, CA, USA) was modified *in vitro*. Briefly, GO-LDL was prepared by incubating N-LDL in 250 mM glucose and 5 µM CuSO_4_ for 7 days under nitrogen at 37°C. After incubation, GO-LDL was dialysed against PBS containing 1 mM EDTA at 4°C to remove glucose and CuSO_4_. The protein content of LDL was quantified with a bicinchoninic acid (BCA) assay kit (Pierce, Rockford, IL). The thiobarbituric acid reactive substances (TBARS) assay was used to assess the lipid peroxidation of LDL using a previously reported method [14]. The degree of glycation was determined by the 2,4,6-trinitrobenzene sulfonic acid assay [15], [16]. Glycated LDL level was determined as the absorbance at 340 nm of relative reduction of the detected amino groups of lysine of LDL. N-LDL and modified LDL were characterised by agarose gel electrophoresis (data not shown) and fluorescence at 360 nm (excitation)/430 nm (emission; Bio-Tek, Winooski, VT, USA), confirming that the advanced glycation end products (AGEs) were similar to those previously reported [14].

**Figure 3:**
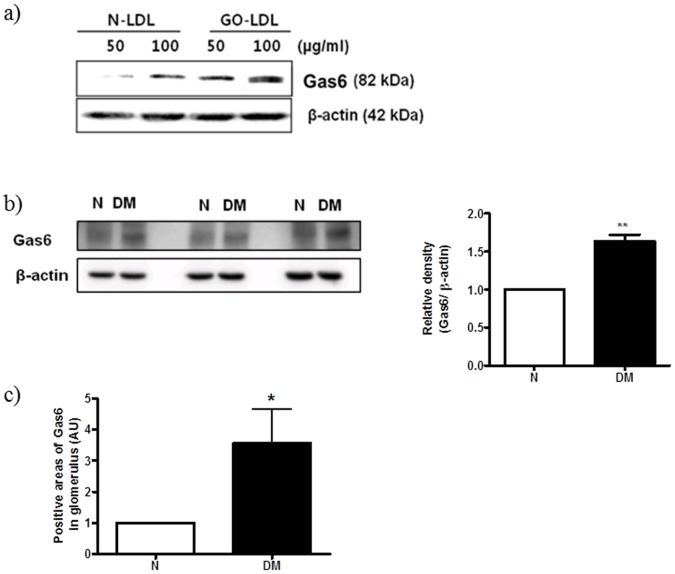
Expression of Gas6 in mesangial cells cultured with GO-LDL and glomeruli from diabetic rats. (a) Cells were treated with GO-LDL (50 or 100 µg/ml) or N-LDL (50 or 100 µg/ml) for 24 h. Western blot data show the significantly increased Gas6 expression in MMCs. Expression of Gas6 protein in the glomeruli of normal and diabetic rats was analysed by Western blotting (b) and immunostaining (c). All data are expressed as the mean ± S.E.M. (n = 4). ^**^P<0.01, ^*^P<0.05 vs. N, respectively.

### Cell Culture and Animal Experiments

MMCs (SV40 MES13) were obtained from the American Type Culture Collection (ATCC, Rockville, MD) and maintained in continuous culture at 37°C/5% CO_2_ using DMEM/F-12 containing 5% FBS and 12 mM HEPES (pH 7.0). Cells were plated into 6-well culture dishes and used for experiments when they reached 80% confluence. Fresh serum-free medium was added to the wells 24 h before experiments. Male 6-week-old Zucker diabetic fatty rats (fa/fa, ZDF, n = 7) and Zucker lean (ZL, n = 7) counterparts (fa/+ or +/+) were purchased from Charles River Laboratories (Waltham, MA, USA) and acclimated for 1 week prior to the study. The two rat study groups were allowed free access to water and food for 14 weeks.

**Figure 4:**
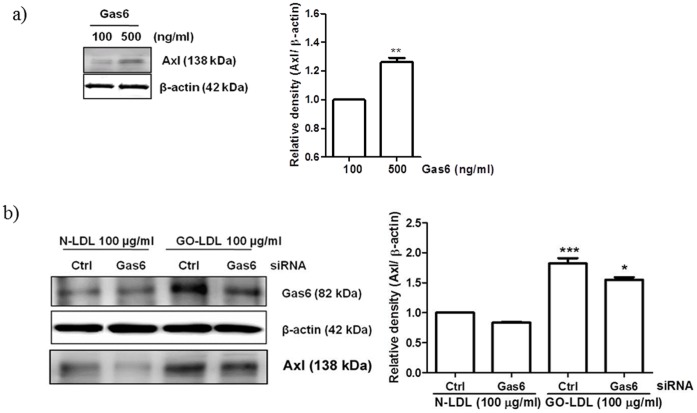
Depletion of Gas6 reduced GO-LDL-induced Axl expression in MMCs. (a) Western blot analysis of Gas6 expression in cells treated with GO-LDL and N-LDL. (b) Cells were transfected with Gas6-specific siRNA or control siRNA and treated with GO-LDL and N-LDL for 24 h. Western blot experiments were conducted as stated in the Methods. The bar graph shows the mean ± S.E.M. of three independent experiments. All data are expressed as the mean ± S.E.M. (n = 3). ^***^P<0.001 vs. N-LDL (100 µg/ml). ^*^P<0.05 vs. GO-LDL with control siRNA.

### Total RNA Preparation and Microarray Experiments

Total RNA in GO-LDL-treated MMCs was isolated and purified (RNeasy Mini kit; Qiagen, Valencia, CA, USA). RNA quality was assured by the A_260_:A_280_ absorbance ratio (>2.0) and the A_260_:A_230_ absorbance ratio (>1.7). The diabetes Oligo-GE Array® (OMM-023) was used for expression profiling (SuperArray Bioscience Corporation, Frederick, MD, USA) in conjunction with the TrueLabeling-AMP linear RNA amplification kit, both of which were used according to the manufacturers’ manuals. Expression profiles from array experiments were analysed using the GE Array expression analysis program.

**Figure 5:**
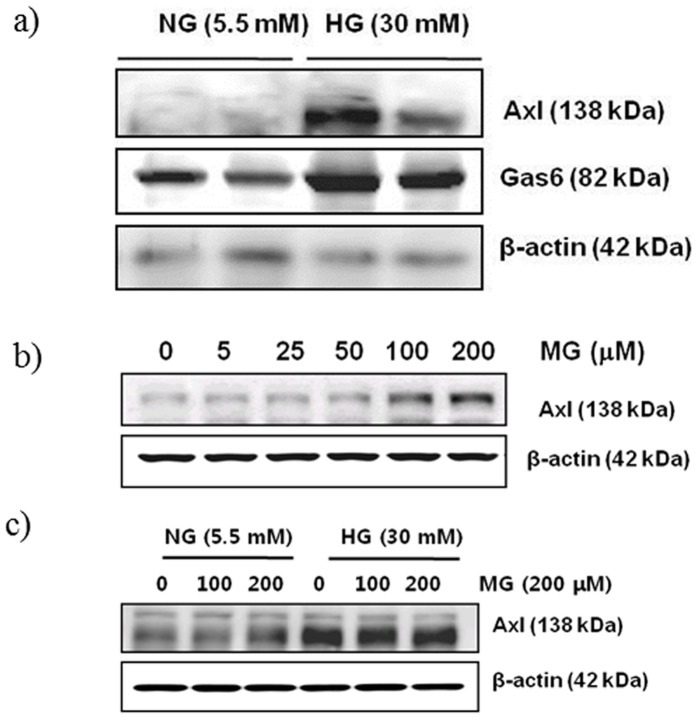
High glucose and MG treatment in MMCs increased Axl and Gas6 expression. (a) Cells were treated with high glucose or NG for 48 h. Western blot experiments were conducted as stated in the Methods. The figure shows a representative blot obtained from three different experiments. (b) Cells were treated with various concentrations of MG for 24 h. (c) Cells were cotreated with high glucose and MG for 24 h, and protein expression was analysed by Western blotting using an Axl-specific antibody.

### Real-time Quantitative PCR for Axl Gene Expression

Real-time qPCR was performed using SYBR Green PCR Master Mix and Chromo4™ Multicolor Real-Time PCR Detection System (Bio-Rad Laboratories). PCR primer sequences were as follows: mouse Axl, 5′-GGAGGAGCCTGAGGACAAAGC-3′ and 5′-T ACAGCATCTTGAAGCCAGAGTAGG-3′; mouse GAPDH, 5′-ACGGCAAATTCAACGGCACAG-3′ and 5′-AGACTCCACGACATACTCAGCAC-3′. In brief, 1 µg of total RNA, which was primed by particular specific primer with a tail sequence recognised by the universal primer, was used for cDNA synthesis. cDNA was amplified using the mouse Axl-specific reverse and universal primers. Amplification of a single fragment was confirmed by a dissociation curve, and good correlation between the standards and threshold-cycle values was observed. The temperature for annealing was optimised depending on the amplification of true targets.

**Figure 6:**
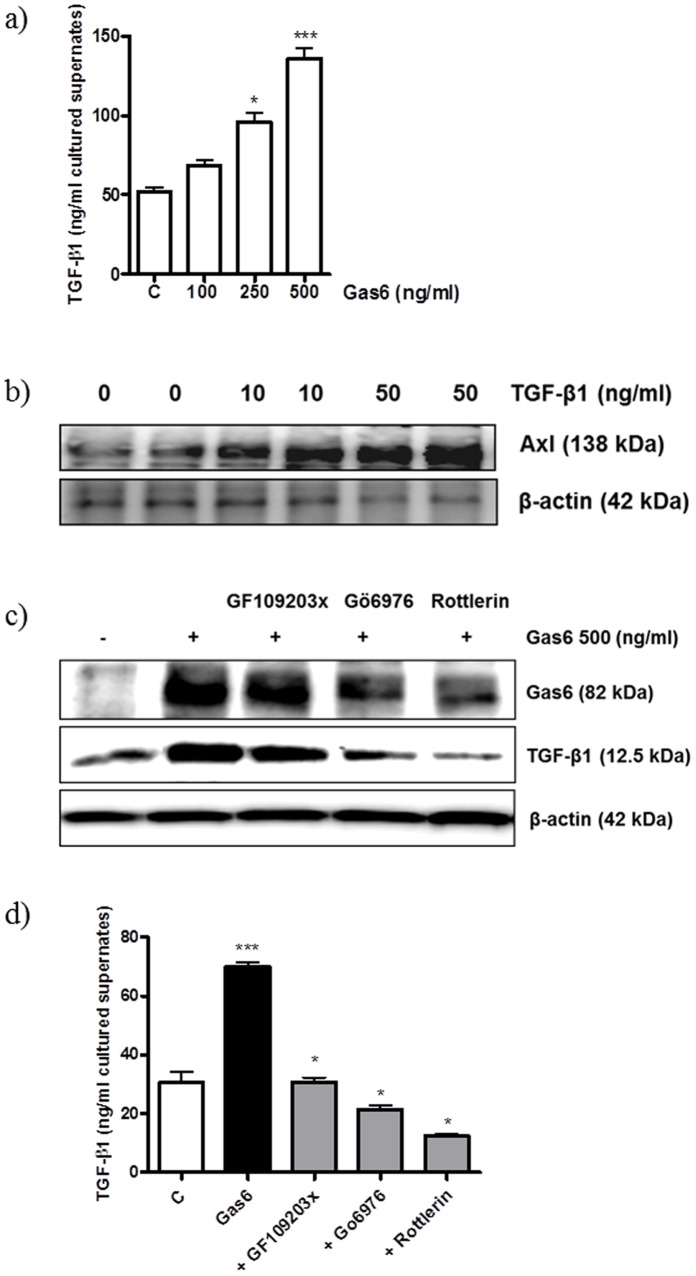
Gas6 treatment increased TGF-β1 secretion, and this secretion induced Axl expression. (a) Cells were treated with Gas6 for 24 h, and cell supernatants were collected for TGF-β1 ELISA analysis. All data are expressed as the mean ± S.E.M. (n = 4). ^***^P<0.001 vs. N-LDL (100 µg/ml). ^*^P<0.05 vs. GO-LDL. (b) Cells were treated for 24 h and analysed by Western blotting using a Axl-specific antibody. Gas6-induced TGF-β1 expression (c) and secretion (d) were inhibited by PKC inhibitors. Cells were pretreated with PKC inhibitors for 30 min and treated with Gas6 for 24 h.

### Western Blot Analysis

Western blotting was performed using a previously described method [14], [17]. Equal amounts of protein (25–50 µg/lane) were subjected to immunoblotting with the indicated antibodies. The antibodies used were as follows: Axl (1∶1000), Gas6 (1∶1000), TGF-β1 (1∶1000), and β-actin (1∶1000). The bound horseradish peroxidase-conjugated secondary antibody was detected using an ECL detection system. Protein expression levels were determined by analysing the signals captured on the nitrocellulose membranes using an image analyser (Las-3000, Fuji photo, Tokyo, Japan).

**Figure 7:**
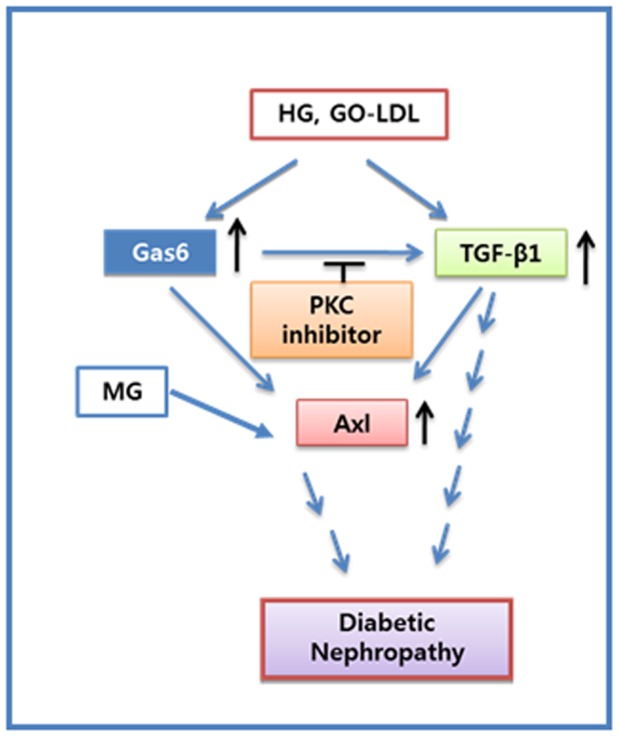
Model of the proposed signalling cascade. High glucose or GO-LDL stimulated Axl expression via Gas6 expression in glomerular mesangial cells. PKC inhibitors inhibited Gas6 induced TGF-β1 expression. Increased Axl expression triggerred the development of DN.

### Morphological Studies

Renal cortices were fixed in 10% formaldehyde and embedded in paraffin, and 4-µm-thick sections were prepared. Positive areas of Axl in glomeruli were measured using NIH Image J software (National Institutes of Health, Bethesda, MD, USA). The cross-section yielding the maximum diameter of the glomerulus was photographed and converted into a digital image. Forty glomeruli were randomly chosen from each rat kidney. For Axl (1∶1000) and Gas6 (1∶500) immunohistochemistry, the deparaffinised sections were hydrated and treated with 1% H_2_O_2_ in methanol. Sections were incubated with anti-Axl antibody for 2 h at room temperature using a standard manual immunoperoxidase procedure with streptavidin-peroxidase (LSAB™ 2 kit, Dako). After straining, the immunofluorescence of Axl (1∶200) in MMCs was observed by a fluorescence microscope (Olympus BX51) equipped with an Olympus DP 70 camera.

### Gas6 Knockdown by siRNA

Predesigned Gas6 siRNAs against mouse Gas6 (sc-35451) and control scrambled siRNA (sc-37007) were purchased from Santa Cruz Biotechnology (Santa Cruz, CA, USA). MMCs were transfected with double-stranded siRNAs (40 nmol/ml) for 6 h into siRNA transfection medium according to the manufacturer’s protocol (Lipofectamine Invitrogen Life, Grand Island, NY, USA) and recovered in fresh media containing 5% FBS overnight. The depletion of Gas6 protein expression was confirmed by Western blot using an anti-Gas6 antibody.

### Quantikine Mouse TGF-β1 Immunoassays (ELISA Assay)

After 80% confluence was reached, the medium was replaced with serum-free medium for 24 h under Gas6 (100–500 ng/ml). Supernatant fractions were harvested. Following activation of TGF-β1 by treatment with 1 N HCl (0.1 ml/0.5 ml of conditioned media) for 10 min at room temperature, 0.1 ml of 1.2 N NaOH/0.5 M HEPES were added. Quantikine mouse TGF-β1 ELISA was performed according to the manufacturer's protocol (R&D Systems, Inc., Minneapolis, MN, USA), and expression levels were normalised to that of total protein. Medium alone without cells incubated under the same conditions was used as blank control for ELISA.

### Statistical Analysis

The results are expressed as means ± S.E.M. of multiple experiments. Paired Student’s t-tests or ANOVA with Tukey’s multiple comparison tests using PRISM software (Graph Pad, San Diego, CA) were used to compare the two groups. A p value <0.05 was considered statistically significant.

## Results

### Upregulation of Axl Expression by GO-LDL in MMCs

To evaluate the modification levels of LDL, we measured peroxidation and glycation levels and the degree of AGE formation in GO-LDL and N-LDL. Levels of TBARS, which are lipid peroxidation markers, were significantly increased in GO-LDL (78.03±4.72 mmol MDA/mg protein) compared to those in N-LDL (5.16±1.33 mmol MDA/mg protein). The degree of glycation was assessed by measuring the amount of lysine residues remaining after modification. The amount of modified-lysine in GO-LDL (∼2-fold) was significantly increased compared with that in N-LDL (Fig. 1B). The measurement of fluorescent material as a marker for AGE formation to detect the effect of test samples on the Maillard reaction was performed according to a previous reported protocol. Relative fluorescence was increased in GO-LDL compared to that in N-LDL (Fig. 1C).

To uncover novel pathways regulated by GO-LDL, we performed gene expression profiling in MMCs by using microarrays specific for diabetes signalling pathways (Fig. 1D). Follow-up validation of our microarray data demonstrated that GO-LDL specifically increased Axl expression compared to N-LDL in MMCs. Figure 2A and b show that GO-LDL increased Axl mRNA and protein expression in MMCs using Western blot and immnuocytochemistry. Next, to check the *in vivo* relevance of our *in vitro* study, we analysed Axl expression in glomeruli from diabetic rats. Diabetic glomeruli exhibited a significant 3-fold increase in Axl expression relative to the control (Fig. 2C).

### Gas6 Expression was Increased in the Glomeruli of ZDF Rats and in MMCs Cultured with GO-LDL

To ascertain whether the upregulation of Gas6, a ligand of Axl, in the glomeruli of diabetic rats and in MMCs cultured with GO-LDL is linked to diabetic conditions, Gas6 protein expression was determined by Western blotting and immunostaining. MMCs were cultured with GO-LDL or N-LDL for 48 h. Our Western blot analyses revealed a significant 2-fold increase in Gas6 protein levels in GO-LDL (100 µg/ml)-treated MMCs compared with the levels in N-LDL (100 µg/ml)-treated cells (Fig. 3A). Next, we isolated glomeruli from the kidneys of ZDF (20-week old) or ZL rats, and Western blot analysis revealed increased Gas6 protein in the glomeruli of ZDF rats compared with that in the glomeruli of ZL rats (Fig. 3B). Immunostaining revealed that the positive area of Gas6 in glomeruli was also increased (Fig. 3C). Thus, we demonstrated that GO-LDL induced Axl expression (Fig. 2A and 2B), and Gas6 levels were increased in the glomeruli of diabetic rats and in MMCs treated with GO-LDL (Fig. 3).

### Knockdown of Gas6 Inhibits GO-LDL-induced Axl Expression in MMCs

To further verify that exposure to Gas6 is linked to the upregulation of Axl in MMCs, cells were exposed to Gas6 for 24 h. As shown as Figure 4A, 500 ng/ml Gas6-treated cells exhibited greater Axl expression than 100 ng/ml Gas6-treated cells. To determine whether GO-LDL-induced Gas6 expression induced Axl expression, RNA interference assays were used. Gas6 expression was silenced by transfecting MMCs with Gas6-specific siRNA (Fig. 4B, upper panel line 4). Gas6 silencing in MMCs significantly reduced GO-LDL-induced Axl expression (Fig. 4B, lower panel line 4).

### Expression of Axl in MMCs Cultured in Simulated Diabetic Conditions

To assess the expression of Axl in MMCs cultured under diabetic conditions, cells were grown in high glucose (30 mM) medium for 48 h and analysed by Western blotting. As expected, Axl expression was increased in high glucose-grown cells compared to that in normal glucose (5.5 mM)-grown cells (Fig. 5A). MMCs were also treated with various concentrations of methylglyoxal (MG, 0–200 µM), which is one of the most important glycation agents for the formation of AGEs. MG increased Axl expression in a dose-dependent manner in MMCs (Fig. 5B). Next, to examine the synergic effects of glucose and MG, cells were simultaneously treated with high glucose and MG. As shown as Figure 5C, no synergistic effects were observed on Axl expression.

### Molecular Mechanism of GO-LDL-induced Axl and TGF-β1 Expression

TGF-β1 is one of the key molecules in DN. It has been shown that GO-LDL increases TGF-β1 expression. To check whether GO-LDL-induced Gas6 expression can also increase TGF-β1 expression in MMCs, we examined TGF-β1 levels in the cell culture supernatants. ELISA analysis revealed a 3-fold increase in TGF-β1 levels in the culture supernatant of MMCs treated with Gas6 (Fig. 6A). This suggests that GO-LDL can upregulate Axl expression by increasing TGF-β1 levels through Gas6, which in turn can increase Axl expression through its own receptor.

We next sought to elucidate the molecular signalling pathway by which modified LDL mediates TGF-β1 expression. We treated MMCs with various PKC inhibitors such as GF109203X (a specific PKC inhibitor), Gö6976 (a specific PKCα and -β inhibitor), and rottlerin (a PKCδ inhibitor). Rottlerin most strongly inhibited Gas6-induced TGF-β1 expression as determined by Western blot analysis (Fig. 6C, second panel). A TGF-β1 ELISA was also performed using the culture medium. Secreted TGF-β1 levels were increased in the Gas6-treated cell culture medium, and PKC inhibitor pretreatment, especially rottlerin, decreased this expression (Fig. 6D).

## Discussion

Much data support the hypothesis that GO-LDL is involved in the pathogenesis of atherosclerosis and diabetes. However, the mechanisms underlying these findings remain to be elucidated. In this study, we first examined the changes of Axl gene expression induced by GO-LDL in MMCs using Oligo-GE arrays. Axl gene expression was significantly increased. Axl protein expression was also significantly increased in MMCs treated with GO-LDL and in the glomeruli of diabetic rats. Moreover, Axl expression was also increased in MMCs cultured under diabetic conditions (high glucose or MG treatment). In addition, Gas6 induced TGF-β1 secretion, and this increased TGF-β1 expression induced Axl expression, suggesting that GO-LDL can increase Axl expression via Gas6-induced TGF- β1 upregulation. The PKC-δ inhibitor rottlerin completely blocked Gas6-induced TGF-β1 expression, suggesting the involvement of a PKCδ-mediated signalling mechanism.

Axl/Gas6 signalling has been implicated in several cellular functions including proliferation and the prevention of apoptosis and adipogenesis in various cells [12], [18], [19]. Previous data suggest that Gas6 induces mesangial cell proliferation via the latent transcription factor STAT3, which may be a therapeutic target for the treatment of renal disease [18]. In diabetes, both increased free and protein-bound glucose are known to undergo nonenzymatic and enzymatic modifications that can result in the formation of low-molecular-mass aldehydes such as MG, glyoxal, and glycolaldehyde. These aldehydes form adducts with lysine and arginine residues, resulting in Schiff base formation, Amadori product rearrangements, and AGE formation [20]. GO-LDL has been shown to be increased in diabetes and triggered the progression of DN. The present study showed that GO-LDL directly increased Axl protein and mRNA expression in MMCs. In addition, cells cultured in diabetic conditions such as high glucose or MG treatment exhibited increased levels of Axl protein.

Gas6 is a novel growth factor for mesangial cells that is posttranslationally activated by C-carboxylation in the presence of vitamin K, and streptozotocin-treated Gas6-knockout mice exhibit less glomerular hypertrophy [21]. Gas6 protein expression was increased in MMCs cultured with GO-LDL in a dose-dependent manner. In addition, depleting Gas6 using siRNA significantly reduced GO-LDL-induced Axl upregulation in MMCs. Thus, GO-LDL could increase Axl through Gas6 singalling. We proposed that this signalling is mediated by TGF-β1 and PKC. Renal TGF-β, a crucial component of the pathogenesis of DN, is significantly upregulated in human DN [22]. In our system, Gas6 increased TGF-β1 levels in MMC supernatant, and TGF-β1 was induced by Axl expression (Fig. 6A and 6B). Gas6 deficiency reduces fibrogenesis and hepatic myofibroblast activation and decreases expression of TGF-β and collagen 1 mRNAs [23]. Activation of the PKC system by hyperglycaemia represents an important mediator of glucotoxicity in DN, and PKC inhibition was proposed to prevent diabetic complications [24], [25]. Diabetes-induced activation of PKCα is crucial for the development of albuminuria, and PKCβ activation is important for mesangial cell expansion, basement membrane thickening, and renal hypertrophy [26]. PKCδ is also linked to the development of pathologies affecting diabetic complications [27]. The simultaneous inhibition of PKC α and β is effective in the treatment of DN in rodents. In the current study, a specific PKCδ inhibitor (rottlerin) completely blocked Gas6-induced TGF-β1 expression (Fig. 6C and 6D). In mice with type 2 diabetes, PKCδ activation is correlated with increased VEGF mRNA translation and kidney hypertrophy [28].

Collectively, these data show that HG or GO-LDL stimulates Axl expression by Gas6 expression in MMCs and PKC inhibitor inhibits Gas6-induced TGF-β1 expression. Thus, increased Axl expression may trigger the development of diabetic nephropathy (Fig. 7).

In conclusion, this study demonstrates for the first time that GO-LDL mediates Axl/Gas6 signalling in MMCs. The expression of Axl was also increased in MMCs cultured under diabetic conditions. Moreover, Axl/Gas6 signalling might be a new therapeutic target for kidney diseases induced by GO-LDL.
